# Insights into the Role of Humic Acid on Pd-catalytic Electro-Fenton Transformation of Toluene in Groundwater

**DOI:** 10.1038/srep09239

**Published:** 2015-03-18

**Authors:** Peng Liao, Yasir Al-Ani, Zainab Malik Ismael, Xiaohui Wu

**Affiliations:** 1 School of Environmental Science and Engineering, Huazhong University of Science and Technology, Wuhan, P.R. China; 2 State Key Lab of Biogeology and Environmental Geology, China University of Geosciences, Wuhan. 430074, P. R. China; 3 Faculty of Engineering, University of AL-Anbar, Al-Anbar Governorate, Iraq

## Abstract

A recently developed Pd-based electro-Fenton (E-Fenton) process enables efficient in situ remediation of organic contaminants in groundwater. In the process, H_2_O_2_, Fe(II), and acidic conditions (~pH 3) are produced in situ to facilitate the decontamination, but the role of ubiquitous natural organic matters (NOM) remain unclear. This study investigated the effect of Aldrich humic acid (HA) on the transformation of toluene by the Pd-based E-Fenton process. At pH 3 with 50 mA current, the presence of HA promoted the efficiency of toluene transformation, with pseudo-first-order rate constants increase from 0.01 to 0.016 as the HA concentration increases from 0 to 20 mg/L. The HA-enhanced toluene transformation was attributed to the accelerated thermal reduction of Fe(III) to Fe(II), which led to production of more hydroxyl radicals. The correlation of the rate constants of toluene transformation and HA decomposition validated hydroxyl radical (**·**OH) as the predominant reactive species for HA decomposition. The finding of this study highlighted that application of the novel Pd-based E-Fenton process in groundwater remediation may not be concerned by the fouling from humic substances.

BTEX (benzene, toluene, ethylbenzene and xylenes) are generally found in petroleum derivatives such as gasoline^1^. They are typically present in petroleum and natural gas production sites, petrol stations, and other areas with underground storage tanks (USTs) or above-ground storage tanks (ASTs) containing gasoline or other petroleum-related products^2^,^3^. BTEX have been frequently detected in groundwater due to their high solubility under ambient conditions (109–1790 mg/L)^4^. As public hazards, benzene, toluene, ethylbenzene and xylenes in drinking water are regulated by U.S. EPA National Primary Drinking Water Regulations with maximum contaminant levels of 0.005, 1.0, 0.7, and 10.0 mg/L, respectively^3^. In situ remediation technologies are in need for BTEX contaminated groundwater.

Electrochemical processes have attracted increasing interests in groundwater remediation as they are simple to manipulate to perform versatile chemical reactions *in situ*^5^,^6^,^7^,^8^. Electro-Fenton (E-Fenton) processes, such as peroxi-coagulation (PC)^9^, electrochemical peroxidation (ECP)^10^ and anodic Fenton treatment (AFT)^11^, are widely reported in the literature of wastewater treatment^12^. However, application of E-Fenton processes in groundwater remediation is limited because it is costly and technically complicated to deliver O_2_ and maintain appropriate pH in the subsurface environments. Moreover, regarding the supply of Fe(II), sacrificial iron anode can be effective but it may produces excess amount of iron sludge^11^,^13^. To overcome the limitation of applying E-Fenton process in subsurface remediation, we have recently developed a novel Pd-based E-Fenton process^14^. As illustrated in Figure S1, the process was performed in a modified three-electrode system, which contains a mixed metal oxides (MMO) anode, an iron cathode, and another MMO cathode (the composition was the same as MMO anode). A feature of the process compared with previous studies with iron anodes is the use of iron as the cathode, and the electrochemical control of Fe(II) production via in situ pH manipulation. Under acidic condition (eqs. 1–3), Pd catalyzes the in situ production of H_2_O_2_ from the combination of electrochemically generated H_2_ and O_2_^5^,^6^,^14^,^15^. Using an iron cathode, Fe(II) is generated in situ from the corrosion of iron electrode under acidic condition (eq. 4) accompanied by H_2_ production (eq. 2). The local acidic condition, which is required for the Pd catalyzed H_2_O_2_ production and release of Fe(II) from the cathode, can be artificially developed by partitioning the current between the iron and the spatially separated MMO cathode, which enables more H^+^ produced at the anode than OH^−^ produced at the iron cathode. Fe(II), H_2_O_2_ and acidity can thus be produced simultaneously in the region near the iron cathode, initiating Fenton reactions with hydroxyl radicals to oxidize organic contaminants. (eqs. 5, 6) Due the balance of electrons in the electric loop, neutral effluent is attained when groundwater passes through the second cathode^6^,^14^,^15^. Our recent results show that this improved Pd-based E-Fenton process can efficiently transform methyl *tert*-butyl ether (MTBE) in artificial groundwater with representative concentrations of inorganic compositions^14^. However, the role of ubiquitous natural organic matter (NOM) on the efficiency of the Pd-based E-Fenton process has not been characterized yet and remains a knowledge gap for field application of this process in groundwater remediation.
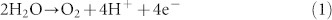

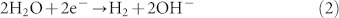

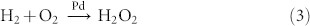

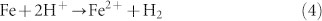

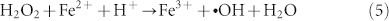

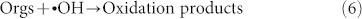


The effects of NOM on contaminants transformation by Fenton and Fenton-based processes have been studied extensively, but the results in the literature are sometimes conflicting^16^,^17^,^18^,^19^,^20^,^21^,^22^,^23^. Some proposed that NOM had a negative^16^ or insignificant impact^17^,^18^ on contaminants transformation because the substrates bound to NOM become less reactive and NOM blocks the attack by hydroxyl radicals (**·**OH). Others noted that NOM accelerated the cycling of Fe(III)/Fe(II), which resulted in enhanced efficiency of decontamination^19^,^20^,^21^. Kang et al.^22^ reported that NOM acted as electron shuttle enhancing the production of H_2_O_2_ and Fe(II), and Page et al.^23^ suggested that the reduced HA could direct produced **·**OH under oxic conditions. The Pd-based E-Fenton process also involves heterogeneous reactions on Pd particles and electrodes, which may be subject to fouling by NOM. The controversial results in literature and the mechanistic complexity of the process pose the need of examining the role of NOM in well controlled experiments that simulate the specific treatment processes.

The primary objective of this study is to evaluate the effect of humic acid, a representative NOM, on the Pd-based E-Fenton transformation of toluene, a representative of BTEX in groundwater. The effect of HA was investigated at different pH and currents. Based on the experimental results, mechanistic information of the NOM's role in the Pd-based E-Fenton process was also discussed.

## Results

### Transformation of toluene in the absence and presence of HA

**Figure 1:**
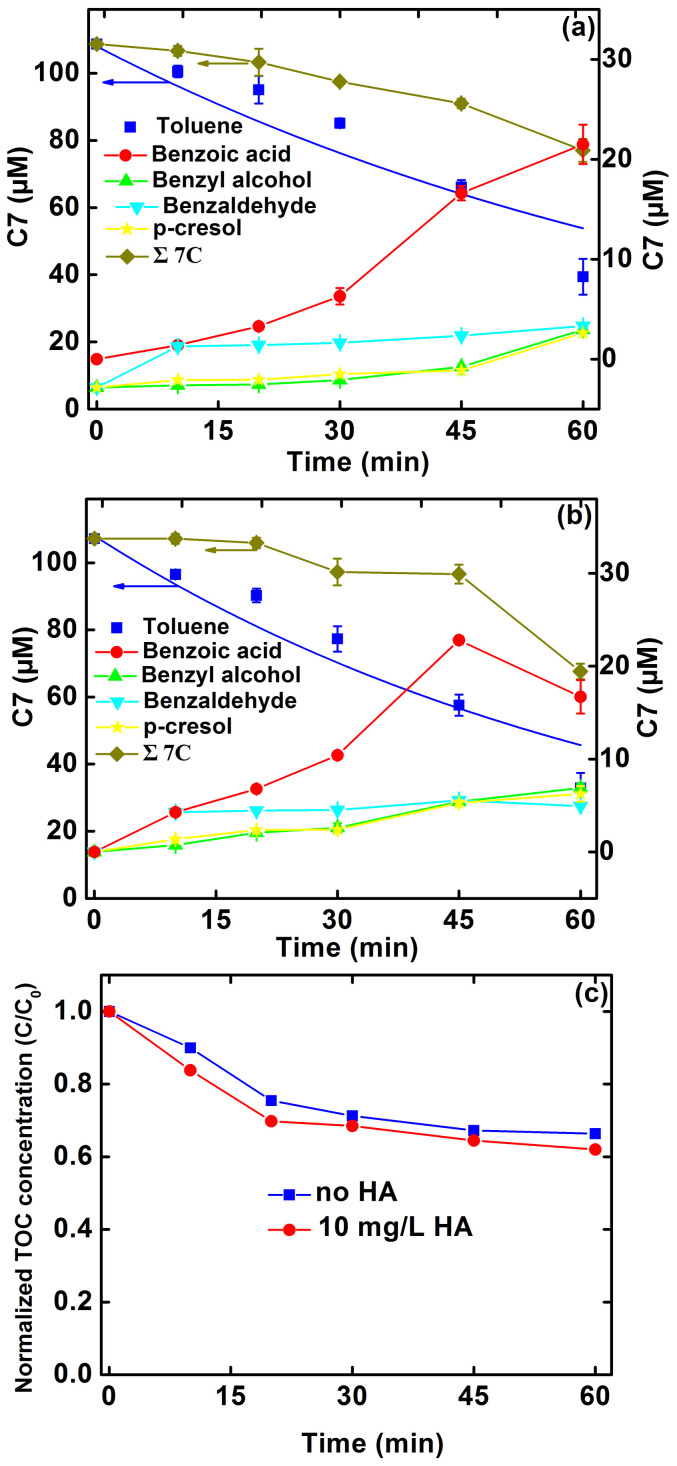
Profiles of toluene transformation by iron cathode in the (a) absence and (b) presence of HA, and (c) mineralization of toluene in the absence and presence of HA. The reaction conditions are based on 10 mg/L initial toluene concentration, initial pH 3.0, 50 mA current, 1 g/L Pd/Al_2_O_3_, 10 mM Na_2_SO_4_ background electrolyte, and 10 mg/L HA if present. Curves refer to pseudo-first-order kinetic fittings. Error bars indicate 95% confidence intervals.

Figure 1 illustrates the transformation profiles of toluene in simulated groundwater in the absence and presence of 10 mg/L HA under conditions of initial pH of 3 and 50 mA current. The control experiment using the iron cathode without electricity or Pd catalyst does not result in any significant transformation of toluene (Figure S2). In the presence of Pd/Al_2_O_3_ and absence of HA (Figure 1a), toluene concentration decreased from 108 to 40 μM within 60 min. Presence of HA in the simulated groundwater improved toluene transformation (Figure 1b) to 32 μM concentration. Another control experiment shows that the adsorption of toluene on HA was minimal (Figure S3), suggesting that the direct redox reaction and complexation-mediated aggregation between toluene and HA are of minor importance^22^. The pseudo-first-order kinetics rate constant for toluene transformation increased from 0.010 min^−1^ in the absence of HA to 0.015 min^−1^ in the presence of 10 mg/L HA (Table 1). The rate constants normalized by Pd concentration (0.05 gPd/L) is 0.2 and 0.3 L/gPd/min in the absence and presence of 10 mg/L HA, respectively, which is in the same level as that reported by our recent work^14^ and one order of magnitude smaller than that reported by Lowry and Reinhard using Pd and H_2_ for TCE hydrodechlorination^24^. The solution pH increased from 3.0 to 3.4 during the course of experiments in the absence and presence of 10 mg/L HA, which is attributed to the consumption of H^+^ by chemical corrosion of the iron cathode (eq. 4).

The primary transformation intermediates in the absence and presence of HA identified are benzoic acid, benzyl alcohol, benzaldehyde, and *p*-cresol, but the variations are different (Figure 1). It should be noted that the benzoic acid could be used to reflect the oxidation of toluene by **·**OH^25^. In the absence of HA, the concentration of benzoic acid gradually increased to about 21.5 μM at 60 min. In the presence of HA, however, benzoic acid reached the maximum concentration of about 22.8 μM at first 45 min followed by a decline. This demonstrates that presence of HA accelerated the generation of **·**OH. Additionally, the relatively lower mass balance of carbon (63%) in the presence of HA compared with that in the absence of HA (71%) implies the production of more **·**OH concentration. The TOC concentrations decreased slightly for transformation of toluene in 60 min (Figure 1c), demonstrating that toluene were mainly transformed to intermediates instead of CO_2_, which corresponds well to the findings of Yuan et al.^15^ The relatively higher TOC removal efficiency in the presence of HA in contrast to in the absence of HA can in part be attributed to the high concentration of **·**OH generated in the process, consistent with the results of carbon mass balance (Figure 1 a, b). Moreover, the transformation of toluene is almost completely inhibited by the addition of 10 mM methanol which is effective in scavenging **·**OH radicals (k**·**_OH_ = 9.7 × 10^8^ M^−1^ s^−1^ 26) (Figure S4). This confirms that HA-enhanced transformation of toluene is predominantly ascribed to the increased production of **·**OH. The underlying mechanism for the enhancement will be discussed later.

### Effect of HA concentration on toluene transformation

**Figure 2:**
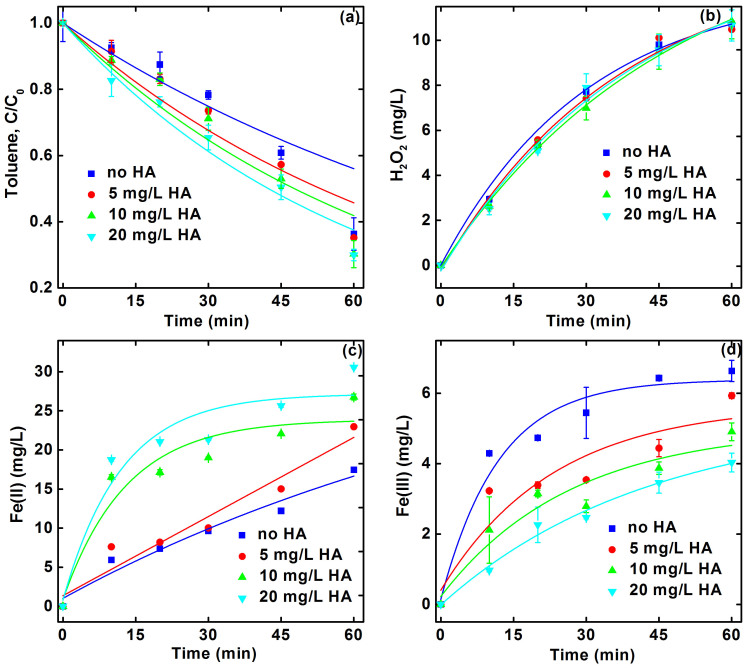
Effect of HA concentration on (a) toluene transformation, (b) H_2_O_2_ production, (c) Fe(II) accumulation, and (d) Fe(III) accumulation. The reaction conditions are based on 10 mg/L initial toluene concentration, initial pH 3.0, 50 mA current, 1 g/L Pd/Al_2_O_3_, and 10 mM Na_2_SO_4_ background electrolyte. Curves refer to pseudo-first-order kinetic fittings. Error bars indicate 95% confidence intervals.

Figure 2a reveals that toluene transformation increased significantly with increasing HA concentration from 0 to 20 mg/L under conditions of pH 3 and 50 mA current, indicating that more HA are advantageous to **·**OH production. The pseudo-first-order decay rate constants increased from 0.010 to 0.016 min^−1^ when the initial HA concentration increased from 0 to 20 mg/L (Table 1). Similar results were also found at pHs of 2 and 5 as well as currents of 20 and 80 mA (Figure S5). As the production of **·**OH is primary derived form the reaction of H_2_O_2_ and Fe(II), the role of HA-enhanced production of **·**OH can be ascribed to (1) facilitated generation of H_2_O_2_ and/or (2) facilitated regeneration of Fe(II).

During the Pd-catalysis process, H_2_O_2_ was mainly produced from the combination of H_2_ and O_2_ on Pd surface (eqs. 1–3)^5^,^6^. When the HA concentration increased from 0 to 20 mg/L, the variation tendency of H_2_O_2_ accumulation is negligible in the presence of Pd/Al_2_O_3_ and absence of Fe(II) (Figure 2b), demonstrating the minimal effect of HA concentration on H_2_O_2_ accumulation. Previous results revealed that the reduced quinone moieties, such as semiquinones and hydroquinones, contained in HA can effectively reduce O_2_ to H_2_O_2_ (eq. 7), and further reduce H_2_O_2_ to **·**OH^27^,^28^,^29^. Other researchers reported that HA can act as an electron-transfer mediator leading to the enhanced production of H_2_O_2_ and **·**OH^23^,^30^. However, the results presented here confirmed that these mechanisms are of less important.
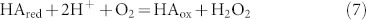


**Figure 3:**
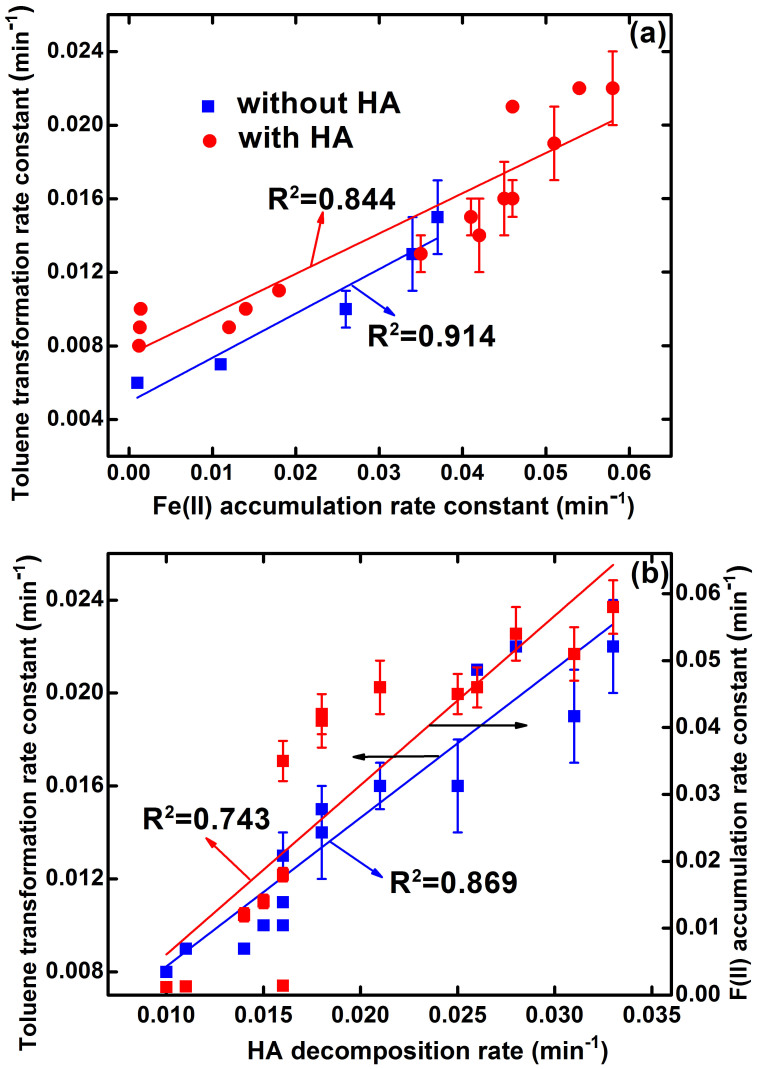
Correlation of (a) toluene transformation rate constants with Fe(II) accumulation rate constants, (b) toluene transformation rate constants with HA decomposition rate constants, as well as HA decomposition rate constants with Fe(II) accumulation rate constants. Error bars indicate 95% confidence intervals.

Then, the effect of HA concentration on Fe(II) accumulation was measured. Figure 3c shows that the accumulated Fe(II) concentration increased remarkably with the increasing HA concentration from 0 to 20 mg/L. The pseudo-first-order kinetics rate constant increased from 0.026 min^−1^ in the absence of HA to 0.035 min^−1^ in the presence of 5 mg/L HA and further to 0.046 min^−1^ in the presence of 20 mg/L HA (Table 1). Similar dependences were also observed at pHs of 2 and 5 and currents of 20 and 80 mA (Figure S6). Note that the regeneration of Fe(II) by iron cathode is difficult because Fe(II) was continuously released from the corrosion of iron cathode under acidic conditions^14^. Hence, the significant augment of Fe(II) concentration can be attributable to the presence of HA.

## Discussion

It is well documented that HA can participate in the oxidation/reduction of iron as a factor controlling the iron speciation^31^,^32^,^33^. Specifically, the quinine and quinine-like compounds (i.e., phenolic and caboxylate moieties) in HA have been recognized to play an important role in the redox cycling of Fe(II) and Fe(III) (eq. 8)^32^,^33^,^34^,^35^,^36^,^37^,^38^. Therefore, it is speculated that HA facilitated the regeneration of Fe(II) from Fe(III) in this study. To further verify this hypothesis, the cumulated concentrations of Fe(III) in the absence and presence of HA was determined (Figure 2d). The results exhibited that the cumulated Fe(III) concentration decreased significantly with increasing HA concentration, and the diminution of Fe(III) was consistent with the augmentation of Fe(II). This proves that HA effectively contributed to the regeneration of Fe(II). Similar results were obtained in the literature^36^,^37^, where HA acted as a catalyst for the thermal reduction of Fe(III) to Fe(II) in natural aquatic system.




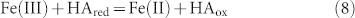




During the Pd-based E-Fenton process, the concentration of Fe(II) generated by iron cathode under acidic conditions is crucial to contaminants transformation in groundwater^14^. In the absence of HA, decreasing solution pH and current is beneficial for toluene transformation and Fe(II) accumulation (Table 1), which is coincided with previous results^14^. A good correlation between toluene decay rate constants and Fe(II) accumulation rate constants was obtained (Figure 3a), proving that the importance of Fe(II) in groundwater for the Pd-based E-Fenton transformation of toluene. In the presence of HA, both toluene transformation rate constants and Fe(II) accumulation rate constant are higher than that in the absence of HA. Interestingly, the toluene transformation rate constants in the presence of HA still linearly correlated with the Fe(II) accumulation rate constants (Figure 3a). This further validates that the transformation of toluene is Fe(II)-dependent and the presence of HA accelerates the accumulation of Fe(II).

**Figure 4:**
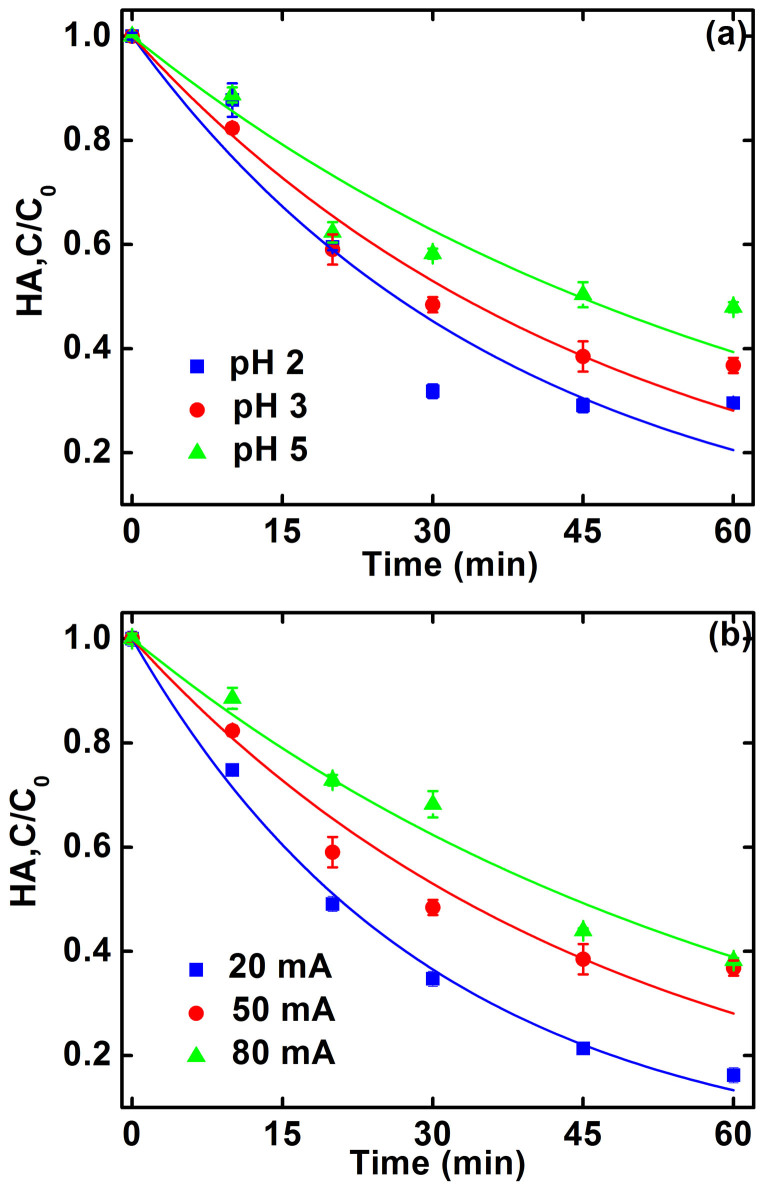
Effects of (a) pH and (b) current on HA decomposition. Unless otherwise specified, the reaction conditions are based on 10 mg/L initial toluene concentration, 10 mg/L HA, initial pH 3.0, 50 mA current, 1 g/L Pd/Al_2_O_3_, and 10 mM Na_2_SO_4_ background electrolyte. Curves refer to pseudo-first-order kinetic fittings. Error bars indicate 95% confidence intervals.

Toluene transformation is accompanied with the decomposition of HA. The decomposition of HA at different solution pHs and currents is presented in Figures 4 and S7, showing pseudo-first-order decomposition kinetics (Table 1). Control experiments show that the decomposition was insignificant without electricity or Pd catalyst (Figure S8). The decomposition of HA increased with decreasing the current and pH (Table 1). This trend is approximately consistent with the transformation of toluene (Figures 2a and S5). Good correlation between toluene transformation rate constants and HA decomposition rate constants (Figure 3b) indicates that **·**OH was mainly responsible for HA decomposition, which agrees with the findings in the literature^38^,^39^,^40^. The reaction of HA with **·**OH could result in the release of bioavailable low molecular weight (LMW) acids^38^,^39^,^40^. It is noted that the enhancement of toluene transformation in the presence of 20 mg/L HA was less significant compared to that in the presence of 10 mg/L HA, while the accumulation of Fe(II) in the presence of 20 mg/L HA was obviously higher than that in the presence of 10 mg/L HA. This phenomenon could be mainly attributed to the competition of HA with toluene for **·**OH, which is supported by the fact that HA decomposition in the absence of toluene was much higher than that in the presence of toluene (Figure S9). It is worthy of notation that there is a good linear correlation between HA decomposition rate constants and Fe(II) accumulation rate constants (Figure 3b). This further verifies the predominant role of HA on increasing Fe(II) regeneration.

**Figure 5:**
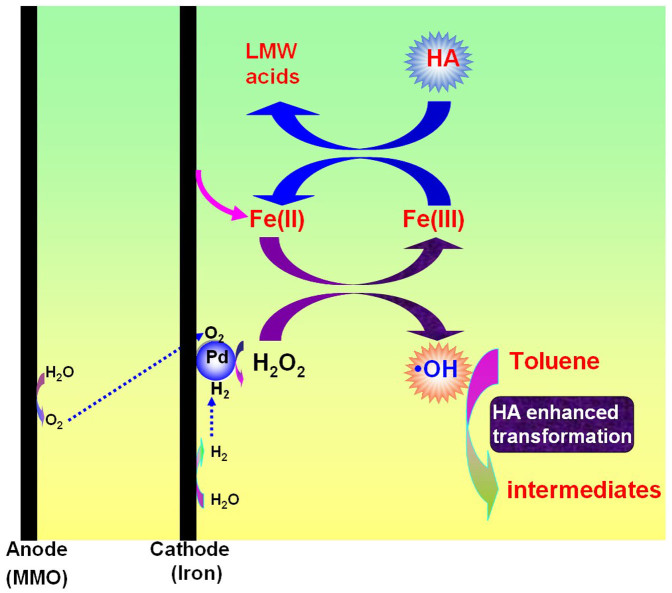
Mechanisms of HA-enhanced transformation of toluene.

In summary, the presence of HA in groundwater was found to significantly enhance the regeneration of Fe(II) from Fe(III), leading to the production of more **·**OH for toluene transformation in Pd-based E-Fenton process (Figure 5). This finding also throws light on the effect of HA on the recycling of Fe(III)/Fe(II) redox in traditional E-Fenton and Fenton-based processes. Although the role of HA in traditional E-Fenton and Fenton-based processes is ambiguous, there was still a few investigations reporting that HA can act as catalyst to facilitate the redox cycle of Fe(II)/Fe(III) accelerating transformation of contaminants^35^,^37^,^41^,^42^. In addition to enhancing the transformation of contaminants, the simultaneous decomposition of HA could avoid or alleviate the negative impact of HA on the catalytic activity of Pd catalysts^43^. Based on the findings in this study and previous results^6^,^14^, it is anticipated that the Pd-based E-Fenton process has the potential to transform contaminants in actual groundwater.

## Methods

### Chemicals and materials

Toluene (99.9%) was purchased from Duksan Chemistry Co. Ltd. Benzoic acid (99.5%), benzyl alcohol (99%), benzaldehyde (99.5%), and *p*-cresol (99%) were supplied by Aladdin Chemistry Co. Ltd. Humic acid (HA) was obtained from Sigma-Aldrich. Aldrich HA was used because it has been used in myriad studies and has relatively high electron-accepting capacities that are similar to actual groundwater^23^. Palladium on alumina powder (5% wt. Pd, Shanxi Kaida Chemical Ltd), with a particle size of 1.5 to 5 μm, was used as the catalyst. Iron plate (S45C type, Wuhan Steel Processing Co., Ltd) and mixed metal oxides (MMO, IrO_2_ and Ta_2_O_5_ coating on titanium diamond mesh, Shanxi Kaida Chemical Ltd) with dimensions of 4.0 cm length, 2.0 cm width and 1.7 mm thickness were used as the cathode and anode, respectively. Prior to the experiments, the iron electrode was polished with coarse emery cloth, etched by diluted HCl solution (5 wt %), and washed with deionized water. Deionized water (18.2 mΩ·cm) obtained from a Millipore Milli-Q system was used in all the experiments. All the chemicals used in this study were above analytical grade.

### Batch experiments

The similar experimental setup as reported previously^14^ is used for toluene transformation at ambient temperature. An MMO mesh and an iron plate were respectively used as the anode and cathode with 40 mm spacing in parallel positions. For each test, 500 mL of 10 mg/L toluene solution was transferred into the cell, and 10 mM Na_2_SO_4_ and 1 g/L Pd/Al_2_O_3_ were attained by the addition of specific masses of Na_2_SO_4_ and Pd/Al_2_O_3_ powder. 10 mM Na_2_SO_4_ was used as the background electrolyte because it commonly exists in shallow groundwater and exerts negligible influence on mechanism analysis^5^. Moreover, our previous study revealed that anionic electrolytes (SO_4_^2−^, Cl^−^, HCO_3_^−^ and NO_3_^−^) in the concentration level less than 10 mM had slight influence on contaminant transformation^5^. Solution pH was adjusted by addition of dilute 1 M H_2_SO_4_ and 1 M NaOH before electrolysis and was not adjusted during the process. The reactor was stirred at 600 rpm using a Teflon-coated magnetic stirring bar. The reaction was initiated by switching on the direct current (DC) power supply (GPC-3060D, Taiwan Goodwill Instrument). The effects of operation parameters including initial solution pH (2, 3, 5), electric current (20, 50, 80 mA), and HA dosage (0, 5, 10, 20 mg/L) were investigated. The electrode potentials for the iron cathode and the MMO anode were −2.1 and 1.1 V (versus SCE) at the total cell potential of 4 V (corresponding to 50 mA current), respectively. The aqueous solution was sampled at pre-determined time intervals and was mixed with 1 mL of methanol to quench further reactions. The concentrations of toluene, iron species, H_2_O_2_, and HA were analyzed. All experiments were carried out in duplicate.

For the analysis of the transformation intermediates, the reactor containing 500 mL of deionized water was purged with N_2_ gas for 30 min to remove CO_2_ before the addition of reactants. Both of the initial concentrations of toluene and HA were set at 10 mg/L. A constant electric current of 50 mA and pH of 3.0 was applied on the cell. About 2 mL of aqueous solution was taken out at predetermined time intervals for analysis of toluene, benzoic acid, benzyl alcohol, benzaldehyde, and *p*-cresol. The solution was filtrated through a 0.45-μm micropore membrane and was then immediately mixed with 1 mL of methanol to quench further reactions. All the experiments were carried out in duplicate.

### Chemical analysis

Toluene, benzoic acid, benzyl alcohol, benzaldehyde, and *p*-cresol concentrations were analyzed using an LC-15C HPLC (Shimadzu) equipped with a UV detector and an XDB-C18 column (4.6 × 50 mm). The mobile phase used a mixture of acetonitrile and water (60:40, v/v) at 1 mL/min, with the detection wavelength at 210 nm. The detection limits for all compounds were 0.1 mg/L. The concentration of total Fe(II) was determined at 510 nm using the 1,10-o-phenanthroline analytical method after dissolving the sample by 1 M HCl. Fluoride was added to avoid the interference of Fe(III) in the determination of Fe(II) dye^44^. Total iron concentration was measured after reducing Fe(III) to Fe(II) by hydroxylamine hydrochloride^44^. The concentration of total Fe(III) was determined by subtracting Fe(II) concentration from total iron concentration. H_2_O_2_ concentration was determined at 405 nm by a spectrometer (UV-1800 PC, Shanghai Mapada Spectrum Instrument Co., Ltd) after coloration with TiSO_4_^45^. The total organic carbon (TOC) concentration was detected by a TOC analyzer (TOC-L CPH, Shimadzu). HA concentration was also measured by spectrometer at 254 nm.

## Supplementary Material

Supplementary Information Supplementary Information Insights into the Role of Humic Acid on Pd-catalytic Electro-Fenton Transformation of Toluene in Groundwater

## Figures and Tables

**Table 1:** Rate constants for toluene degradation, Fe(II) accumulation, and HA decomposition at different conditions^a^

Variation parameters		Toluene transformation		Fe(II) production		HA decomposition	
		k_1_ (min^−1^) b	R^2^	k_1_ (min^−1^)^b^	R^2^	k_1_ (min^−1^)^b^	R^2^
PH 2	HA = 0	0.013 ± 0.002	0.899	0.034 ± 0.003	0.967	—	—
	HA = 5 mg/L	0.014 ± 0.002	0.922	0.042 ± 0.003	0.985	0.018 ± 0.002	0.991
	HA = 10 mg/L	0.021 ± 0.000	0.988	0.046 ± 0.003	0.981	0.026 ± 0.003	0.922
	HA = 20 mg/L	0.022 ± 0.000	0.996	0.054 ± 0.004	0.978	0.028 ± 0.001	0.952
PH 3	HA = 0	0.010 ± 0.001	0.901	0.026 ± 0.002	0.940	—	—
	HA = 5 mg/L	0.013 ± 0.001	0.939	0.035 ± 0.003	0.954	0.016 ± 0.000	0.956
	HA = 10 mg/L	0.015 ± 0.001	0.924	0.041 ± 0.004	0.869	0.018 ± 0.001	0.956
	HA = 20 mg/L	0.016 ± 0.001	0.967	0.046 ± 0.004	0.883	0.021 ± 0.002	0.966
PH 5	HA = 0	0.006 ± 0.000	0.948	0.001 ± 0.000	0.986	—	—
	HA = 5 mg/L	0.008 ± 0.000	0.987	0.0012 ± 0.000	0.978	0.010 ± 0.002	0.902
	HA = 10 mg/L	0.009 ± 0.000	0.996	0.0013 ± 0.000	0.965	0.011 ± 0.001	0.956
	HA = 20 mg/L	0.010 ± 0.000	0.970	0.0014 ± 0.000	0.964	0.016 ± 0.000	0.864
20 mA	HA = 0	0.015 ± 0.002	0.936	0.037 ± 0.002	0.983	—	—
	HA = 5 mg/L	0.016 ± 0.002	0.935	0.045 ± 0.003	0.973	0.025 ± 0.002	0.995
	HA = 10 mg/L	0.019 ± 0.002	0.952	0.051 ± 0.004	0.972	0.031 ± 0.000	0.996
	HA = 20 mg/L	0.022 ± 0.002	0.969	0.058 ± 0.004	0.978	0.033 ± 0.001	0.973
50 mA	HA = 0	0.010 ± 0.001	0.901	0.026 ± 0.002	0.940	—	—
	HA = 5 mg/L	0.013 ± 0.001	0.939	0.035 ± 0.003	0.954	0.016 ± 0.000	0.956
	HA = 10 mg/L	0.015 ± 0.001	0.924	0.041 ± 0.004	0.869	0.018 ± 0.001	0.956
	HA = 20 mg/L	0.016 ± 0.001	0.967	0.046 ± 0.004	0.883	0.021 ± 0.002	0.966
80 mA	HA = 0	0.007 ± 0.000	0.942	0.011 ± 0.001	0.972	—	—
	HA = 5 mg/L	0.009 ± 0.000	0.992	0.012 ± 0.001	0.985	0.014 ± 0.000	0.975
	HA = 10 mg/L	0.010 ± 0.000	0.995	0.014 ± 0.001	0.909	0.015 ± 0.001	0.954
	HA = 20 mg/L	0.011 ± 0.000	0.973	0.018 ± 0.001	0.946	0.016 ± 0.000	0.958

^a^Unless otherwise specified, the reaction conditions are based on 10 mg/L initial toluene, 1 g/L Pd/Al_2_O_3_, pH 3, 50 mA current, and 10 mM Na_2_SO_4_ background electrolyte.

^b^k_1_ is pseudo-first-order reaction rate constant. Pseudo-first-order reaction kinetics is given by ln(C_t_/C_0_) = −*k_1_*t + b, where *k*_1_ is the first-order rate constant (min^−1^), *t* is the reaction time (min), b is a constant, and C_0_ and C_t_ are the concentrations (μM) at times of t = 0 and t = t, respectively.
