# Supplementary material for: Insights into the Role of Humic Acid on Pd-catalytic Electro-Fenton Transformation of Toluene in Groundwater
Source: Sci Rep. 2015 Mar 18;5:9239. doi: 10.1038/srep09239 (PMC4363861; doi:10.1038/srep09239)
Supplement: Supplementary Information — Insights into the Role of Humic Acid on Pd-catalytic Electro-Fenton Transformation of Toluene in Groundwater [file srep09239-s1.docx]

**Supplementary Information**

**Insights into the Role of Humic Acid on Pd-catalytic Electro-Fenton Transformation of Toluene in Groundwater**

Peng Liao^*,a,b^, Yasir Al-Ani^b,c^, Zainab Malik Ismael^b,c^, Xiaohui Wu^*,a^

The supplementary information (SI) contain 9 figures.


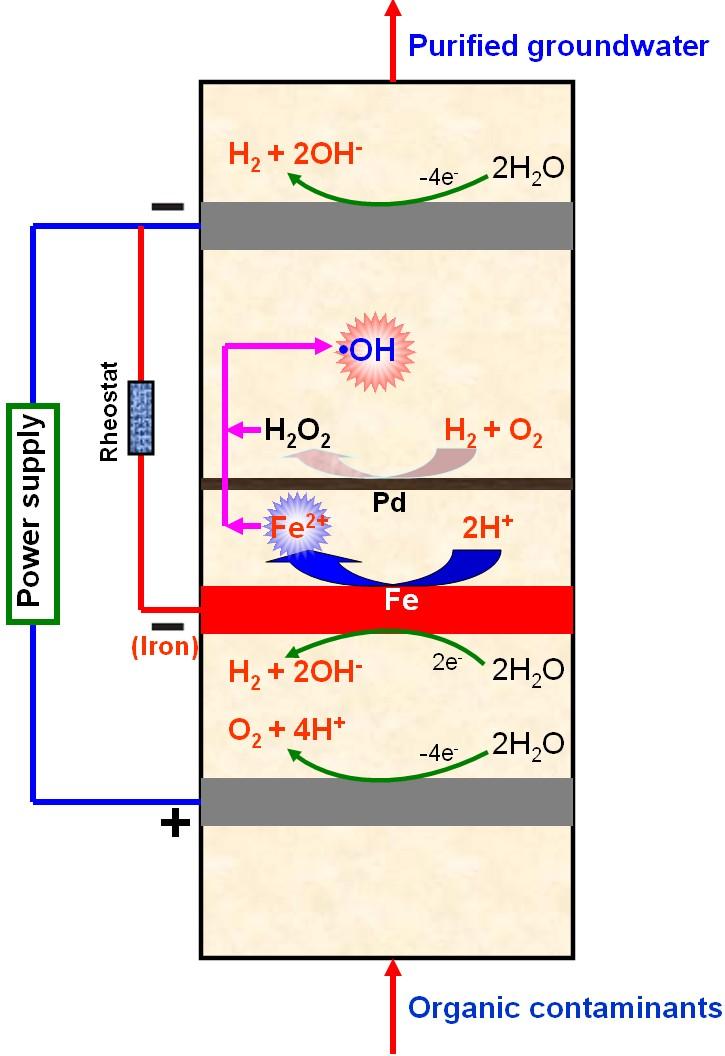


**Figure S1. Conceptual model of novel Pd-based E-Fenton process.** The process was performed in a modified three-electrode system, which contains a mixed metal oxides (MMO) anode, an iron cathode, and another MMO cathode (the composition was the same as MMO anode).


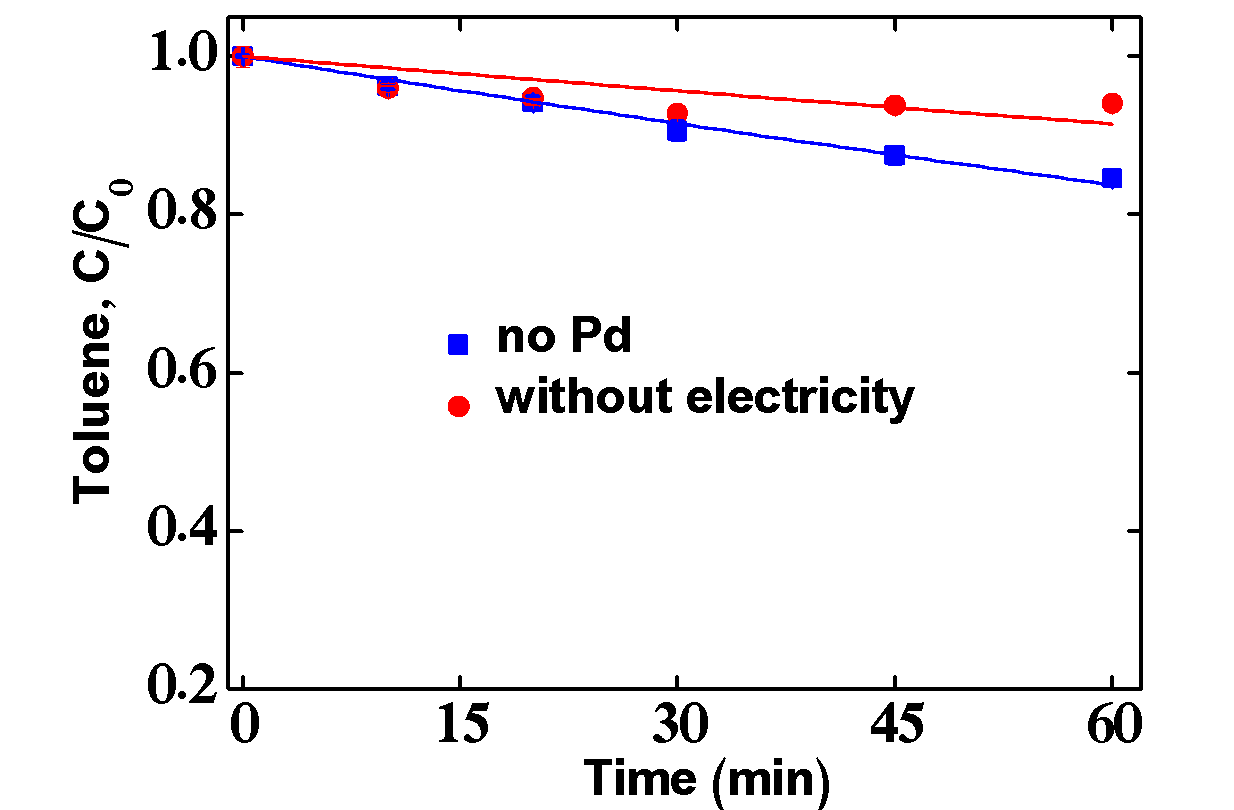


**Figure S2.** Transformation of toluene without electricity or Pd catalyst. The reaction conditions are based on 10 mg/L initial toluene concentration, initial pH 3.0, and 10 mM Na_2_SO_4_. Error bars indicate 95% confidence intervals.


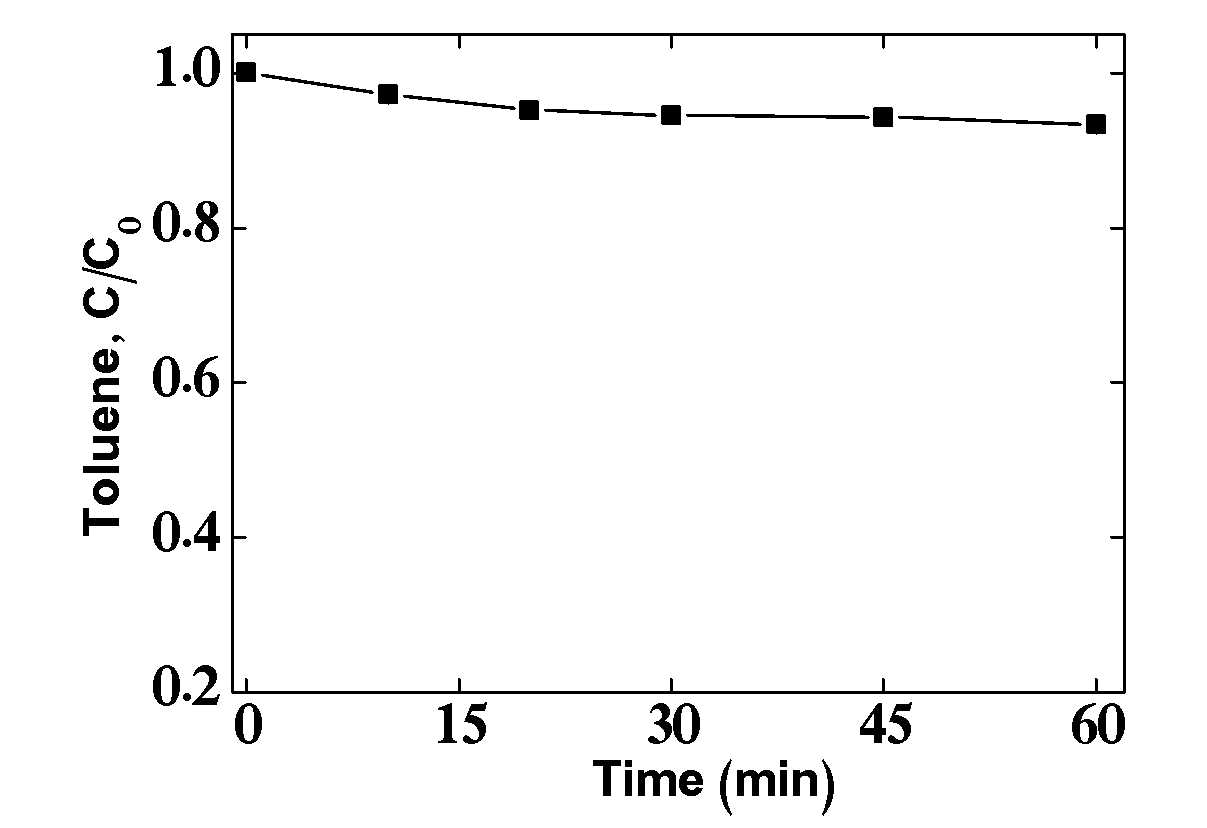


**Figure S3.** Adsorption of toluene on HA. The reaction conditions are based on 10 mg/L initial toluene concentration, 10 mg/L HA, and initial pH 3.0, and 10 mM Na_2_SO_4_. Error bars indicate 95% confidence intervals.


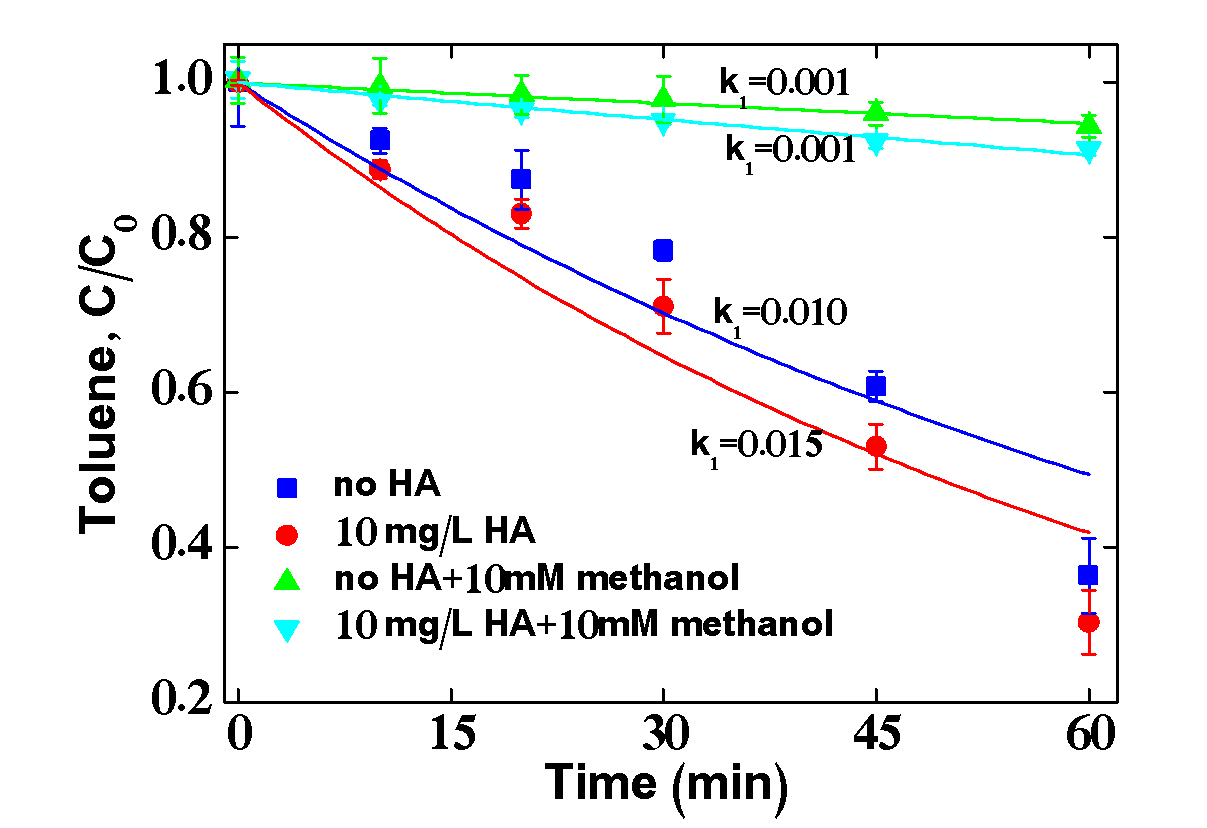


**Figure S4.** Effect of radical scavenging agents on toluene transformation. The reaction conditions are based on 10 mg/L initial toluene concentration, initial pH 3.0, 50 mA current, and 10 mM Na_2_SO_4_. Error bars indicate 95% confidence intervals.


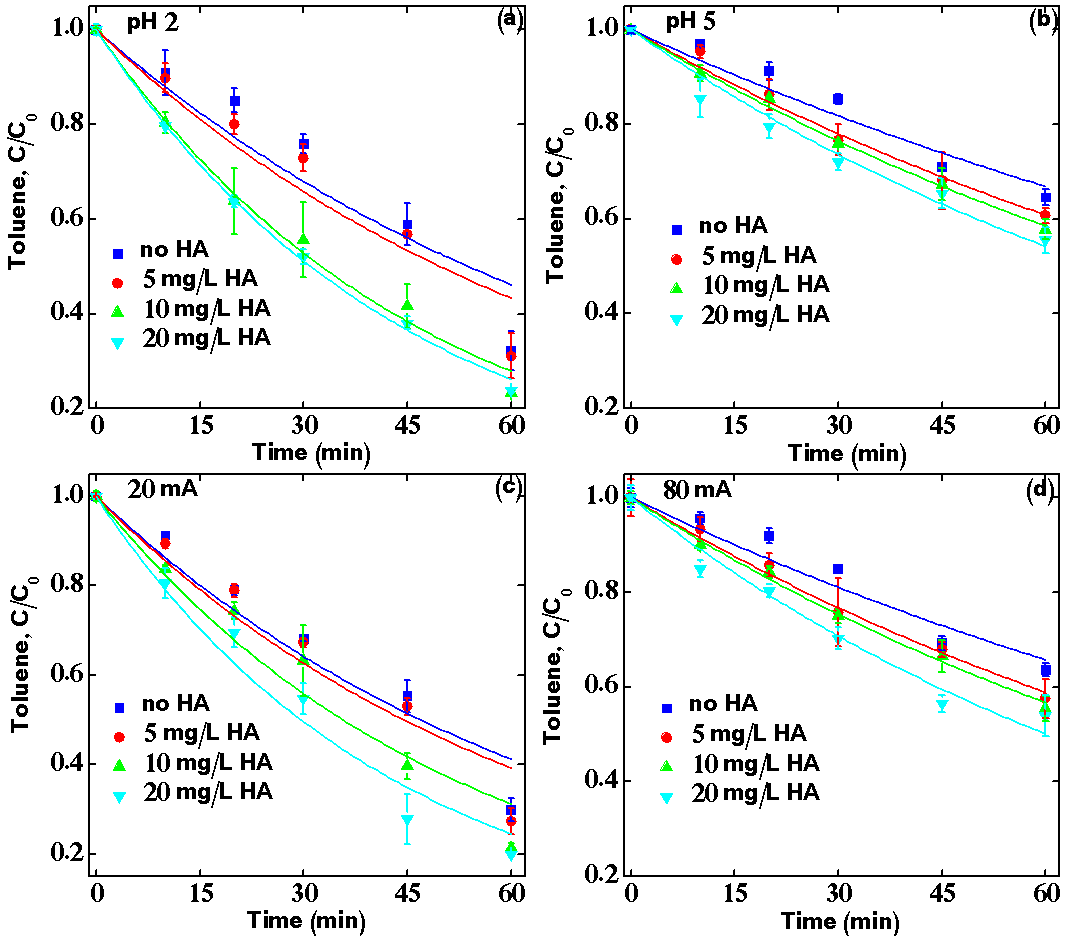


Figure S5. Effect of HA concentration on toluene transformation at (a) pH 2, (b) pH 5, (c) 20 mA current, and (d) 80 mA current. Unless otherwise specified, the reaction conditions are based on 10 mg/L initial toluene concentration, initial pH 3.0, 50 mA current, 1 g/L Pd/Al_2_O_3_, and 10 mM Na_2_SO_4_ background electrolyte. Curves refer to pseudo-first-order kinetic fittings. Error bars indicate 95% confidence intervals.


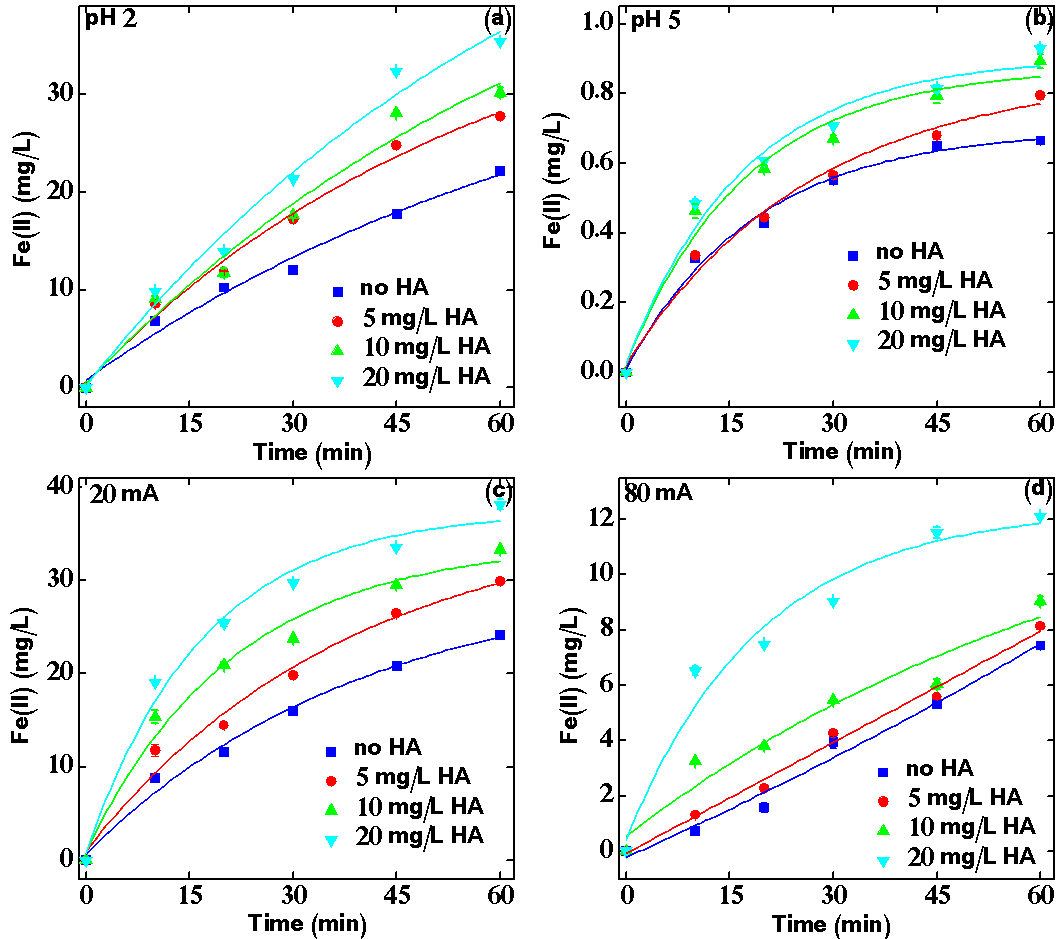


Figure S6. Effect of HA concentration on Fe(II) accumulation at (a) pH 2, (b) pH 5, (c) 20 mA current, and (d) 20 mA current. Unless otherwise specified, the reaction conditions are based on 10 mg/L initial toluene concentration, initial pH 3.0, 50 mA current, 1 g/L Pd/Al_2_O_3_, and 10 mM Na_2_SO_4_ background electrolyte. Curves refer to pseudo-first-order kinetic fittings. Error bars indicate 95% confidence intervals.


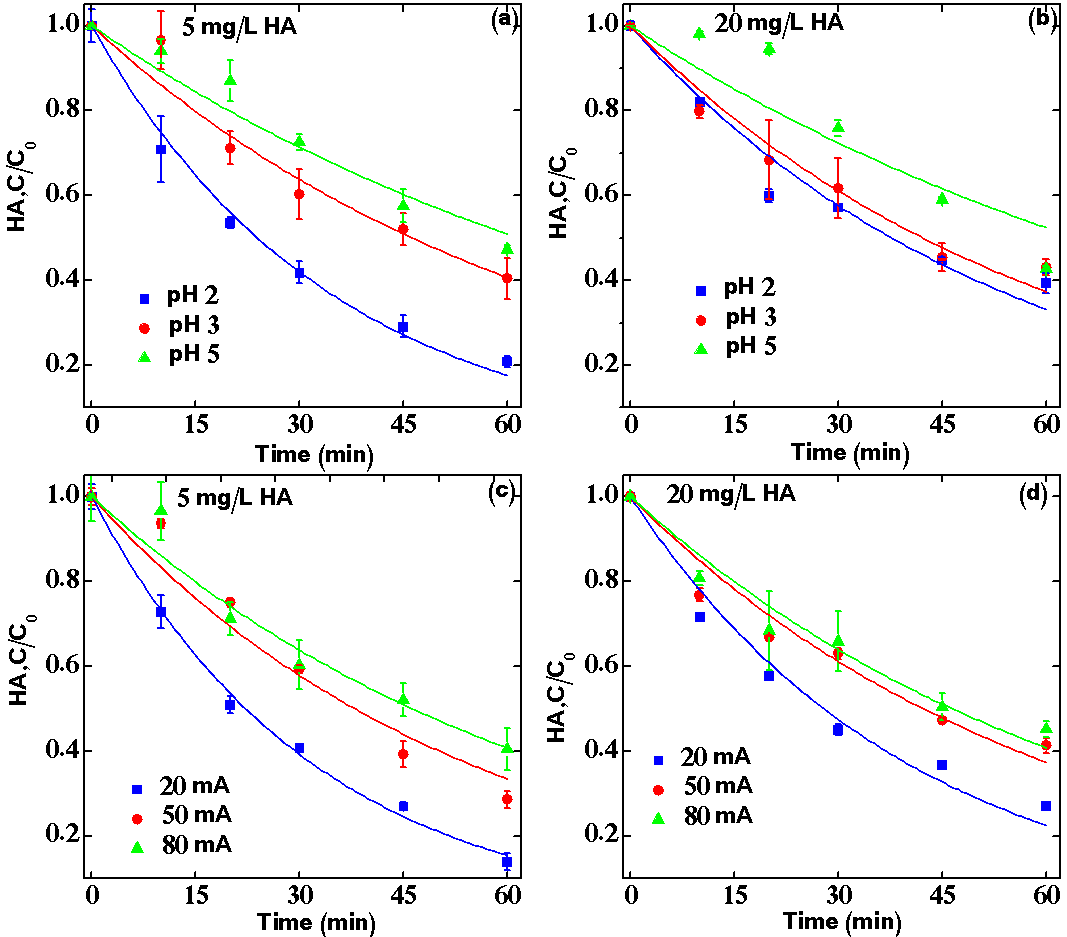


Figure S7. Effect of (a, b) pH and (c, d) current on HA decomposition. Unless otherwise specified, the reaction conditions are based on 10 mg/L initial toluene concentration, initial pH 3.0, 50 mA current, 1 g/L Pd/Al_2_O_3_, and 10 mM Na_2_SO_4_ background electrolyte. Curves refer to pseudo-first-order kinetic fittings. Error bars indicate 95% confidence intervals.


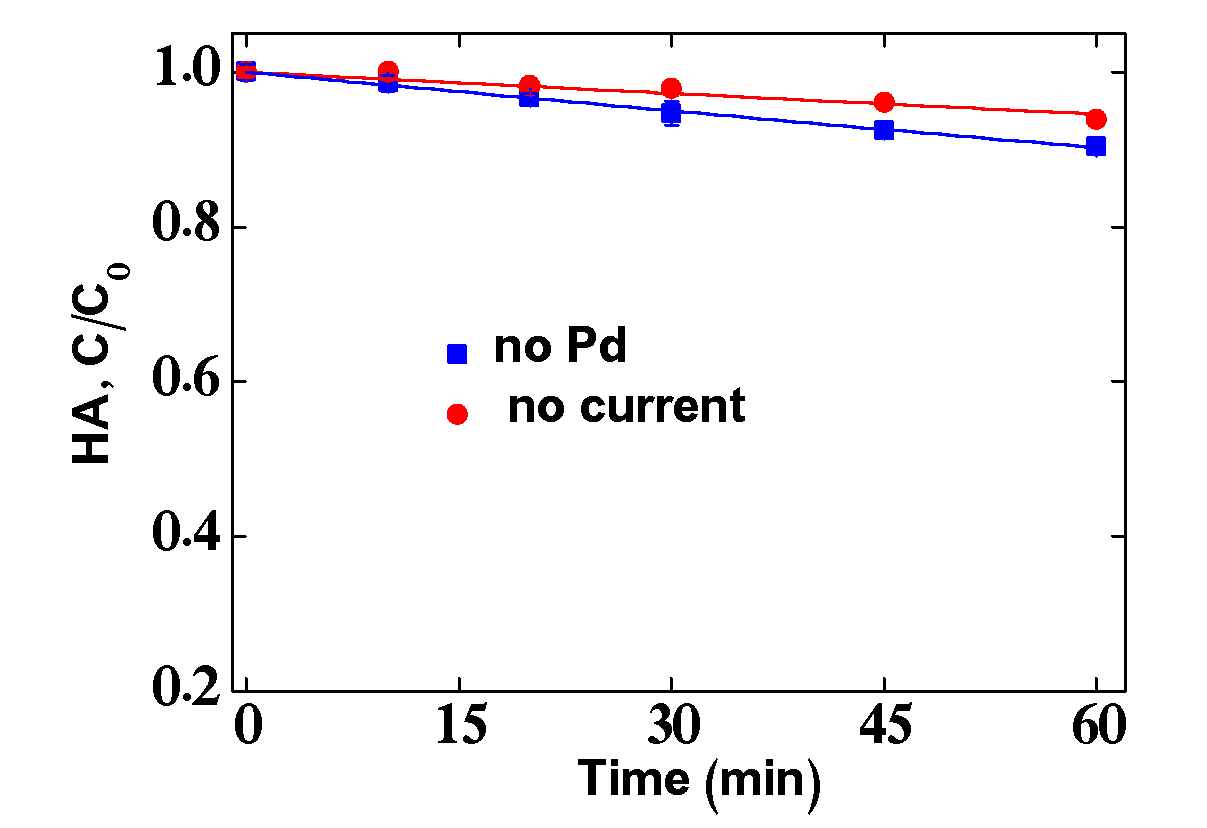


**Figure S8.** Decomposition of HA without Pd catalyst or current. The reaction conditions are based on 10 mg/L initial HA concentration, initial pH 3.0, and 10 mM Na_2_SO_4_. Error bars indicate 95% confidence intervals.


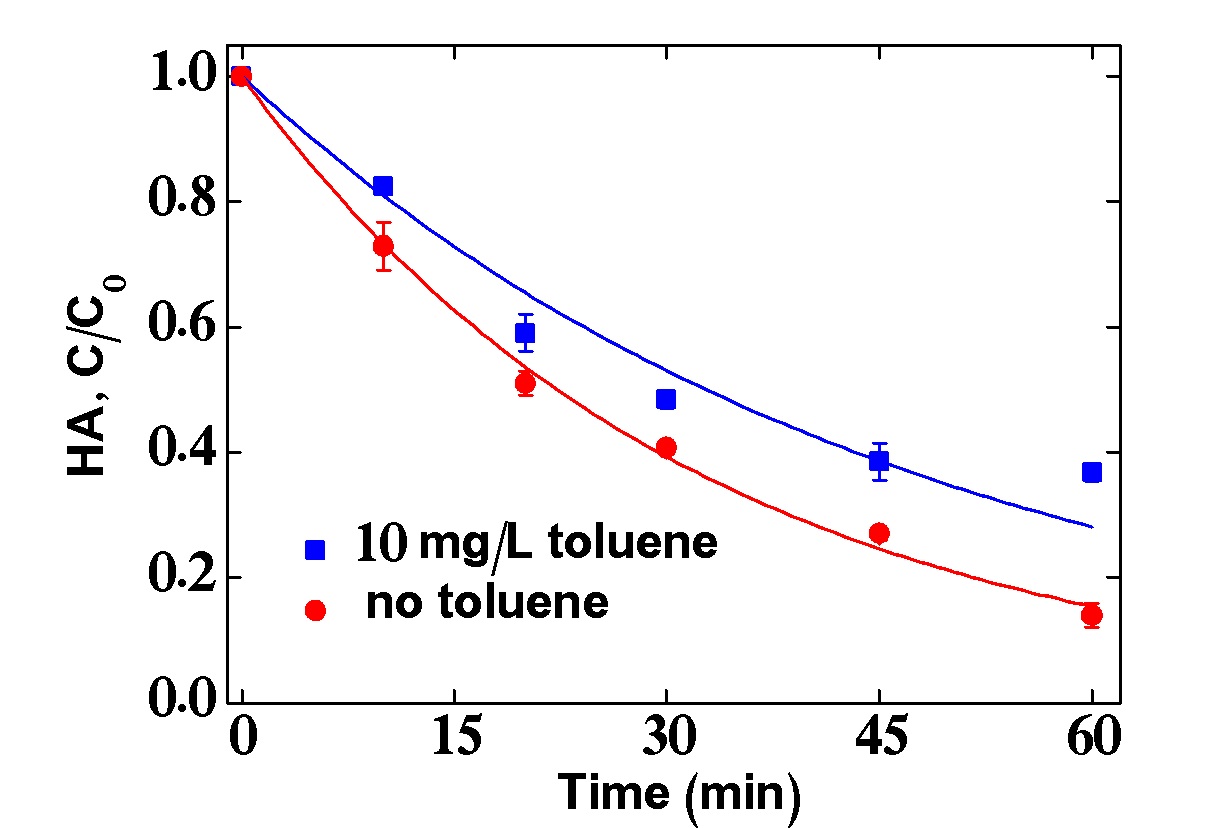


**Figure S9.** Decomposition of HA in the absence and presence of toluene. The reaction conditions are based on 10 mg/L initial HA concentration, initial pH 3.0, 50 mA current, and 10 mM Na_2_SO_4_. Error bars indicate 95% confidence intervals.
